# Modulation of rabbit muscle sarcoplasmic reticulum Ca^2+^-ATPase activity by novel quercetin derivatives

**DOI:** 10.2478/intox-2013-0001

**Published:** 2013-03

**Authors:** Dušan Blaškovič, Petronela Žižková, Filip Držík, Jana Viskupičová, Miroslav Veverka, Ľubica Horáková

**Affiliations:** 1 Institute of Experimental Pharmacology and Toxicology, Slovak Academy of Sciences, Dúbravská cesta 9, 841 01 Bratislava, Slovak Republic; 2 EUROFINS BEL/NOVAMANN Ltd, 940 02 Nove Zámky, Slovak Republic

**Keywords:** calcium pump, scavenging, flavonoids, antioxidants

## Abstract

Sarcoplasmic reticulum Ca^2+^-ATPase (SERCA) is the pump crucial for calcium homeostasis and its impairment results in pathologies such as myopathy, heart failure or diabetes. Modulation of SERCA activity may represent an approach to the therapy of diseases with SERCA impairment involvment. Quercetin is flavonoid known to modulate SERCA activity. We examined the effect of nine novel quercetin derivatives on the activity of the pump. We found that 5-morpholinohydroxypoxyquercetin, di(prenylferuoyl)quercetin, di(diacetylcaffeoyl)-mono-(monoacetylcaffeoyl)quercetin and monoacetylferuloylquercetin stimulated the activity of SERCA. On the contrary, monochloropivaloylquercetin, tri(chloropivaloyl)quercetin, pentaacetylquercetin, tri(trimethylgalloyl)quercetin and diquercetin inhibited the activity of the pump. To identify compounds with a potential to protect SERCA against free radicals, we assessed the free radical scavenging activity of quercetin derivatives. We also related lipophilicity, an index of the ability to incorporate into the membrane of sarcoplasmic reticulum, to the modulatury effect of quercetin derivatives on SERCA activity. In addition to its ability to stimulate SERCA, di(prenylferuloyl)quercetin showed excellent radical scavenging activity.

## Introduction

The Ca^2+^-ATPase of sarcoplasmic reticulum (SR) is a particularly abundant protein, and plays a very important role in excitation-contraction coupling. Calcium is released from sarcoplasmic reticulum through calcium-release channels (ryanodine receptors) resulting in muscle contraction. Sarco/endoplasmic Ca^2+^-ATPase (SERCA) pumps calcium back into sarcoplasmic reticulum which leads to muscle relaxation. Changes in the calcium transport have been reported to play a role in a variety of pathological processes, such as vascular diseases, diabetes, myopathy and heart failure (Hovnanian, 2007). In pathological processes that are accompanied by elevated levels of free radicals SERCA becomes inactivated. Generally, therapies aimed on improving contractility are based on increasing the activity of the pump.

Flavonoids, belonging to the group of phytoestrogens, which are rich in fruit and vegetables have been described to modulate SERCA activity (Ogunbayo *et al.*, 2008). Quercetin (Q), the most abundant of plant flavonoids, is an excellent peroxynitrite scavenger (Haenen *et al.*, 1997). It inhibits the release of histamine and proinflammatory cytokines (Park *et al.*, 2008) and induces apoptosis in cancer cells by downregulation of anti-apoptic Bcl2 and upregulation of pro-apoptic Bax protein (Duo *et al.*, 2012). Novel quercetin derivatives were synthesized with the aim to prepare biologically active compounds which enhance antioxidative properties and/or bioavailability of quercetin. In this paper, we examined the influence of novel quercetin derivatives on the activity of SERCA and determined free radical scavenging properties of quercetin derivatives using the DPPH radical scavenging assay.

## Materials and methods

### Synthesis of quercetin derivatives

Novel quercetin derivatives were prepared via selective protection procedures of quercetin and acylation with acylchlorides: The ultimate step consisted of deprotection and column chromatography. Partial alkylation and careful separation from a complex mixture produced naphtoquinone derivative. The dimer of quercetin was obtained by its treatment with a metal salt at 50 °C (patent filed). Structures of quercetin derivatives are shown in Table 1.

**Table 1:** Structures of quercetin and its derivatives tested. 
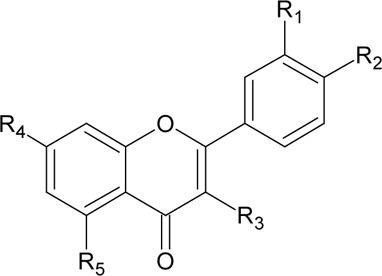

Name	Substituents			
5-morpholinohydroxy propoxyQ	R1=	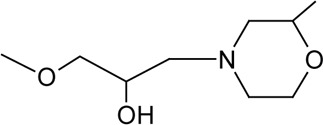	R2-5=	-OH
di(prenylferuloyl)Q	R1,2=	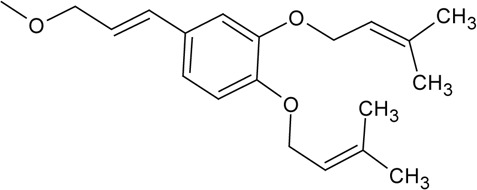	R3-5=	-OH
di(diacetylcaffeoyl)-mono-(monoacetylcaffeoyl)Q	R1=	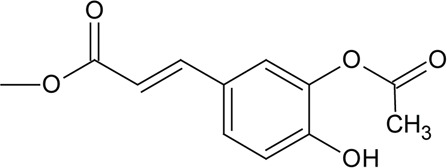	R2,4=	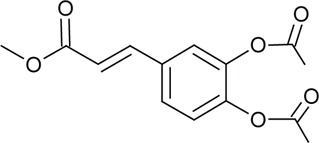
			R5=	-OH
monoacetylferuloylQ	R1=	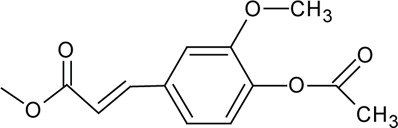	R2-5=	-OH
pentaacetylQ	R1-5=	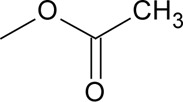		
quercetin	R1-5=	-OH		
tri(trimethylgalloyl)Q	R1-3=	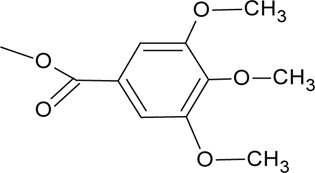	R4,5=	-OH
monochloropivaloylQ	R1=	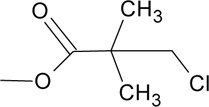	R4-5=	-OH
tri(chloropivaloyl)Q	R1,4,5=	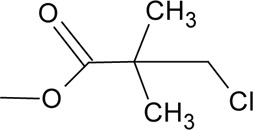	R2,3=	-OH

**Structure**				

diquercetin		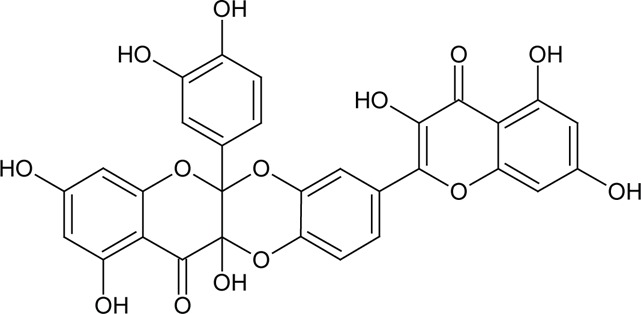		

### Isolation of sarcoplasmic reticulum

SR vesicles were isolated from fast-twitch skeletal muscle of a New Zealand female rabbit (about 2.5 kg) according to (Warren *et al.*, 1974) and modified by (Karlovská *et al.*, 2006).

SR vesicles (1 mg protein per ml) were incubated with quercetin or individual quercetin derivatives at concentrations 10–150 µM for 2 min at 37 °C, pH 7.4

### Ca^2+^-ATPase activity

The activity of sarcoplasmic reticulum Ca^2+^-ATPase was measured by the NADH-coupled enzyme assay according to Warren *et al.* (1974). The sarcoplasmic reticulum vesicles (12.5 µg protein per cuvette) were added to the assay mixture (40 mM HEPES pH 7.2, 0.1 M KCl, 5.1 mM MgSO_4_, 2.1 mM ATP, 0.52 mM phosphoenolpyruvate, 1 mM EGTA, 0.15 mM NADH, 7.5 IU of pyruvate kinase, 18 IU of lactate dehydrogenase) and incubated at 37 °C for 2 minutes. The reaction was started by addition of 1 mM CaCl_2_ to the reaction mixture. The reaction rate was determined by measurement of the absorbance at 340 nm and 37 °C.

### DPPH free radical scavenging assay

To investigate the radical scavenging activity of the quercetin derivatives, the ethanolic solution of DPPH (50 µM) was incubated in the presence of the compound tested (50 µg/ml) at laboratory temperature. The absorbance decrease recorded during the first 75-s interval at 518 nm, was taken as a marker of radical scavenging activity. The decrease in absorbance is expressed in Absorbance Units.

## Results

**Figure 1:**
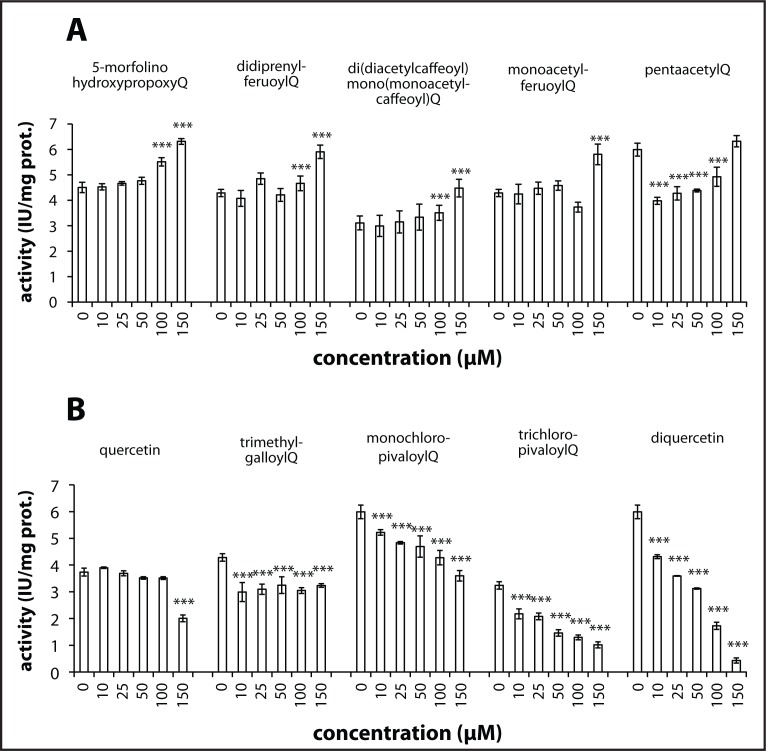
Effect of quercetin or its derivatives on SERCA activity. Values are mean ± SEM and represent three independent experiments with at least three parallels. The statistical analysis of the data was performed using un-paired ANOVA test with Turkey comparison. ***p<0.001, control versus treated SERCA

Quercetin had little effect on SERCA activity up to 100 µM. We observed slight inhibition of SERCA by quercetin at 150 µM (Figure 1B). Figure 1A shows that 5-morpholinohydroxypropoxyQ, di(prenylferuloyl)Q, di(diacetylcaffeoyl)-mono-(monoacetylcaffeoyl)Q and monoacetylferuloylQ stimulated SERCA activity. MonochloropivaloylQ, tri(chloropivaloyl)Q and diquercetin remarkably inhibited SERCA activity (Figure 1B). Tri(trimethylgalloyl)Q and pentaacetylQ inhibited the pump slightly.

In order to determine the radical-scavenging potential of the quercetin derivatives tested, the reactivity toward the stable free radical DPPH was measured by absorbance decrease of ethanol solution of DPPH (50 µM) containing the samples tested (50 µM) at 518 nm (Table 2). Absorbance decrease was determined during the first 75-s interval and compared with the effect of the reference antioxidant quercetin. The antiradical activity of the quercetin derivatives is shown in Table 2 and decreases as follows: monochloropivaloylQ > quercetin > diquercetin > di(prenylferuloyl)Q > tri(chloropivaloyl)Q > di(diacetylcaffeoyl)-mono-(monoacetylcaffeoyl)Q > monoacetylferuloylQ > 5-morpholinohydroxypropoxyQ > pentaacetylQ > tri(trimethylgalloyl)Q.

**Table 2:** DPPH radical scavenging efficiency, lipophilicity of quercetin derivatives and their effect on SERCA activity

Compound	DPPH ▵A(518nm/75s)	logP (lipophilicity)	SERCA activity
5-morpholinohydroxypropoxyQ	0.014 ± 0.002	2.83 ±1.08	↑
di(prenylferuloyl)Q	0.316 ± 0.015	15.48 ±1.04	↑
di(diacetylcaffeoyl)-mono-(monoacetylcaffeoyl)Q	0.0425 ± 0.006	4.13 ± 1.34	↑
monoacetylferuloylQ	0.0395 ± 0.019	3.68 ±1.09	↑
pentaacetylQ	0.0098 ± 0.003	0.15 ±1.37	↔
quercetin	0.3859 ± 0.038	2.07 ±1.31	↓
tri(trimethylgalloyl)Q	0.0083 ± 0.004	7.16 ±1.38	↓
monochloropivaloylQ	0.446 ± 0.045	2.47 ±1.2	↓
tri(chloropivaloyl)Q	0.119 ± 0.023	4.66 ±1.34	↓
diquercetin	0.374 ± 0.057	6.07 ±1.12	↓

individual symbols: ↑-stimulation,↓-inhibition, ↔reaching level of untreated SERCA values for DPPH ▵A(518nm/75s) are expressed as mean ± SEM. Results are mean values ± SD from at least three experiment.

LogP values (octanol-water partition coefficients) indicating lipophilicity and thus the ability of quercetin derivatives to incorporate into the lipid membrane of SR were calculated using ACD/Chemsketch software and are specified in Table 2. LogP values of quercetin derivatives decrease as follows: di(prenylferuloyl)Q > tri(trimethylgalloyl)Q > diquercetin > tri(chloropivaloyl)Q > di(diacetylcaffeoyl)-mono-(monoacetylcaffeoyl)Q > monoacetylferuloylQ > 5-morpholinohydroxypropoxyQ > monochloropivaloylQ > quercetin > pentaacetylQ.

## Discussion

SERCA is crucial for calcium homeostasis and its impairment results in a variety of pathologies. SERCA is a therapeutical target and its 60–80% content in SR (Engelender *et al.*, 1995) makes it a good model for the study of its activity/properties. The aim of this study was to identify quercetin derivatives which modulate activity of SERCA. Novel quercetin derivatives may affect SERCA activity as follows: (i) incorporate into the SR membrane, change both its properties and SERCA activity; (ii) interact directly with amino acid residues of SERCA through non-covalent intractions changing SERCA conformation; (iii) autooxidize and change into reactive forms which may inibit SERCA. SERCA is exposed to increased concentrations of a variety of free radicals such as peroxynitrite, hydrogenperoxide and hydroxyl radicals. Therefore, in addition to studying the effect of quercetin derivatives on SERCA activity, we tested free radical scavenging efficiency of quercetin derivatives using DPPH assay.

Properties of the phospholipid bilayer exert an impact on SERCA activity. It has been shown that change in bilayer thickness, fluidity and head group content of the SR membrane affects the activity of SERCA (Gustavsson *et al.*, 2011; Li *et al.*, 2012). SERCA activity depends on properties of the SR membrane and thus inhibition or stimulation of SERCA by quercetin derivatives may be due to changes of physico-chemical properties of the membrane. MonochloropivaloylQ and tri(chloropivaloyl)Q significantly decreased the activity of SERCA (Figure 1A). The incorporation of pivalate through an ester linkage to a bioactive molecule is an effective and established strategy for engineering a drug with improved systemic delivery of the bioactive molecule (Beaumont *et al.*, 2003). Compared with quercetin, the pivalate-containing quercetin has a higher lipophilicity and is likely to cross lipid biomembranes more efficiently. The LogP values indicate increased lipophilicity of monochloropivaloylQ and tri(chloropivaloyl)Q in comparison to quercetin (Table 2). MonochloropivaloylQ and tri(chloropivaloyl)Q may directly interact with SERCA through halogen bonding (Parisini *et al.*, 2011), which is characterized by non-covalent interaction between halogen-bearing compounds and nucleophiles in protein backbone which contain NH and C=O groups. According to DPPH test monochloropivaloyl Q is better scavenger in comparison to quercetin (Table 2), while tri(chloropivaloyl)Q showed markedly decreased scavenging activity in comparison to quercetin.

5-morpholinohydroxypropoxyQ showed stimulating effect on SERCA. Hydrophilicity of 5-morpholinohydroxypropoxyQ is slightly increased in comparison to quercetin. Thus a change of membrane properties by 5-morpholinohydroxypropoxyQ may not be the factor affecting SERCA activity. Morpholino oxygen in 5-morpholinohydroxypropoxyQ is likely to interact with the amide backbone of SERCA, which may result in its conformational change. The scavenging activity of 5-morpholinohydroxypropoxyQ was found to be negligible.

Interestingly, di(prenylferulolyl)Q has a noticeably increased logP value in comparison to all other quercetin derivatives tested (Table 2), which may indicate its extraordinary lipophilicity. Prenyl groups in di(prenylferuloyl)Q may form hydrophobic interactions with hydrophobic amino acid residues along with hydrogen interaction between oxygen in prenylferulolyl moiety and NH-groups in the protein backbone of SERCA. According to its logP value, di(prenylferuloyl)Q is likely to be the most hydrophobic among all quercetin derivatives tested. Di(prenylferuloyl)Q stimulated SERCA activity and additionally showed scavenging activity comparable to quercetin (Table 2). Thus, di(prenylferuloyl)Q has the potential both to protect SERCA against free radicals and to stimulate SERCA activity.

Diacetylcaffeoyl- and monoacetylferuloyl- groups in quercetin derivatives stimulated the activity of SERCA. Di(diacetylcaffeoyl)-mono-(monoacetylcaffeoyl)Q and monoacetylferuloylQ are more lipophilic in comparison to quercetin. The negligible decrease in DPPH absorbance indicates low scavenging properties of di(diacetylcaffeoyl)-mono-(monoacetylcaffeoyl)Q and monoacetylferuloylQ. Substitution of all hydroxyl groups in the molecule of quercetin resulting in pentaacetylQ caused a slight inhibition of SERCA. The activity of SERCA in the presence of pentaacetylQ reached the level of the control at 150 µM. PentaacetylQ is a poor DPPH scavenger and its logP value is lower than that of quercetin. A low logP value indicate weak lipophilicity of pentaacetylQ, which may thus be unable to interact with the membrane. Tri(trimethylgalloyl)Q inhibited the activity of SERCA. Tri(trimethygalloyl)Q is a lipophilic molecule (logP=7.6) and is likely to integrate into the membrane. Presence of gallic acid moiety, which by itself possesses scavenging activity, failed however to show antioxidative activity when present in tri(trimethylgalloyl)Q.

Quercetin had little effect on SERCA activity in concentrations up to 100 µM. However, we observed inhibition of SERCA by quercetin at 150 µM. The inhibitory effect of quercetin may be explained by its autooxidation. Quercetin is autooxidized in PBS buffer and gives rise to stable semiquinone radicals (Lei *et al.*, 2008; Zhou & Sadik, 2008). The semiquinone radical may donate an electron to molecular oxygen, thus generating superoxide anion. Semiquinone radicals were shown to be cytotoxic (Metodiewa *et al.*, 1999). Products of quercetin oxidation were demonstrated to damage cells through thiol arylation (Boots *et al.*, 2007). The remarkable inhibitory effect of diquercetin may be explained by autooxidation and formation of semiquinones, which may decrease SERCA activity by its oxidation. The increase in inhibition of diquercetin is likely due to increased lipophilicity and/or increased formation of semiquinones in comparison to quercetin as two molecules form diquercetin. Quercetin and diquercetin have similar scavenging activities.

Inhibition of SERCA results in increased intracellular load of calcium within cells, which results in increased endoplasmic reticulum stress and apoptosis (Lam *et al.*, 1993). Diquercetin, tri(chloropivaloyl)Q and monochloropivaloylQ showed considerable inhibition of SERCA. Inhibition of SERCA activity by quercetin derivatives may prove to be beneficial in treatment of cancer by induction of selective apoptosis in cancerous cells. On the contrary, stimulation of SERCA by di(prenylferuloyl)Q, di(diacetylcaffeoyl)-mono-(monoacetylcaffeoyl)Q, monoacetylferuloylQ and 5-morpholinohydroxypropoxyQ may be advantageous in relieving of SERCA inhibition in diseases such as myopathy and cardiovascular diseases.

## Conclusion

SERCA activity modulation and elimination of consequences of oxidative stress has been recognized as an important strategy in the prevention and attenuation of diseases caused by SERCA impairment. In view of the effects of quercetin derivatives on SERCA activity, we believe that further investigations of quercetin derivatives will prove to be beneficial in identifying potential remedies for SERCA involved diseases.
